# Interfacial Confinement Enables Dual Hosting of Sulfur Mustard and Mustard Sulfoxide in NU-1000

**DOI:** 10.1021/acs.langmuir.6c04057

**Published:** 2026-10-10

**Authors:** Farrah S. McCormick, Liangliang Huang

**Affiliations:** † School of Sustainable Chemical, Biological and Materials Engineering, 6187 University of Oklahoma, Norman, Oklahoma 73019, United States; ‡ Materials Science and Engineering Program, University of Oklahoma, Norman, Oklahoma 73019, United States

## Abstract

Zirconium-based metal–organic frameworks have emerged as promising materials for the capture and detoxification of chemical warfare agents, but the molecular mechanism by which a hierarchical MOF differentiates a hydrophobic reactant from its oxidized product remains incompletely understood. Here, we use molecular dynamics simulations, grand-canonical Monte Carlo adsorption calculations, and periodic density functional theory to examine the dual-hosting behavior of NU-1000 toward sulfur mustard (HD) and mustard sulfoxide (HDO). Rather than treating NU-1000 only as an adsorbent or oxidation catalyst, we resolve how its internal micropore, mesopore, and c-pore interfaces impose distinct confinement environments on reactant and product species. Density functional theory (DFT) calculations show that HD binds most favorably in the c-pore region, with a binding energy of approximately −12.5 kcal/mol, and similarly favorable stabilization in confined micropore environments, whereas substantially weaker binding is observed in the mesopore. Molecular dynamics simulations also reveal sharply different pore residence patterns: HD localizes predominantly in micropores, occupying this region for ∼80% of the trajectory, while HDO samples larger pore environments more broadly, with ∼45% mesopore and ∼34% c-pore occupancy. This pore-selective behavior persists under mixed HD/HDO coadsorption conditions, demonstrating that reactant–product differentiation is intrinsic to the NU-1000 pore network rather than an artifact of isolated single-component simulations. Radial distribution functions further show that HD is stabilized by dispersion-dominated confinement and weak halogen–hydrogen contacts, whereas HDO is governed by more localized, orientation-dependent interactions with hydrated zirconium node sites. These interaction differences produce a pronounced transport asymmetry, with HDO exhibiting a self-diffusion coefficient of 10.6 ± 2.11 × 10^–10^ m^2^/s compared with 0.5 ± 0.09 × 10^–10^ m^2^/s for HD at comparable loading. The results identify NU-1000 as a dual-function molecular host that couples reactant retention with product mobility, providing an interface-level mechanism relevant to selective CWA detoxification and confined transport in hierarchical porous catalysts.

## Graphical abstract

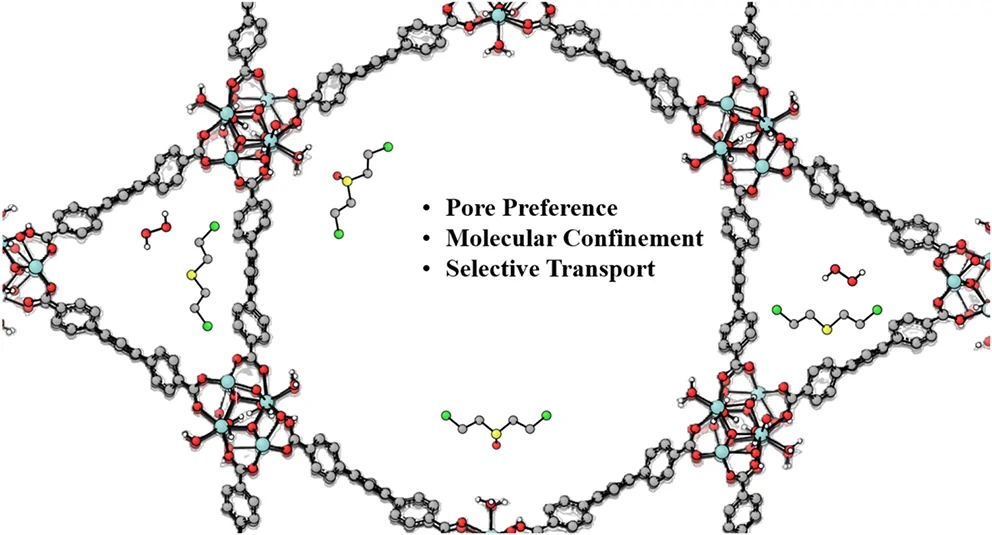

## 1 Introduction

Metal–organic frameworks (MOFs) have gained significant interest in catalysis and separations because their metal nodes, organic linkers, pore architectures, and defect environments can be systematically tuned to control molecular adsorption, transport, and reactivity. The detoxification of chemical warfare agents (CWAs) within stable porous MOF environments is particularly compelling, given the ability of these materials to facilitate hydrolysis and oxidation pathways for nerve and blistering agents.^1^ While computational studies have examined the degradation of nerve agents such as Sarin (GB) and Soman (GD),^1,2^ experimental studies have shown that oxidation of sulfur mustard, bis­(2-chloroethyl) sulfide (HD), can be kinetically enhanced within MOF environments compared to reactions in the absence of a framework.^3–6^ However, molecular-level understanding of HD adsorption, diffusion, and preferential pore residence within hierarchical MOFs remains limited. Computational approaches are well suited to address this gap because they can resolve adsorption sites, diffusion pathways, host–guest interactions, and reactant–product separation mechanisms that are difficult to isolate experimentally.

Mustard gas, bis­(2-chloroethyl) sulfide, is a blistering agent that attacks the human body through the skin, eyes, and respiratory system leading to the destruction of tissue upon exposure often ending in severe blistering, permanent blindness, or death.^7^ Although HD has been internationally banned, continued storage and threat of CWA release remains important concerns. HD can be neutralized through hydrolysis, oxidation, and dehydrohalogenation pathways.^8–10^ However, hydrolysis is kinetically limited by the inherently hydrophobic nature of sulfur mustard. As a result, oxidation has emerged as a favorable detoxification pathway, particularly within MOF environments where tunable pore structures and internal interfaces can influence adsorption, transport, and reactivity. Many studies have focused on photocatalytic oxidation, where MOFs facilitate the excitation of O_2_ to singlet oxygen (^1^O_2_), enabling selective transformation of HD.^4–6,11–13^ Other studies have examined oxidants such as hydrogen peroxide (H_2_O_2_), a common cleaning agent, for a rapid decontamination of CWAs.^14–21^ However, excessively rapid or uncontrolled oxidation can lead to overoxidation and formation of the undesired toxic sulfone product (HDO_2_).^4,22,23^ Controlling this selectivity is therefore critical, as partial oxidation to sulfoxide (HDO) is significantly less toxic and therefore preferred. Experimental studies suggest that factors such as pore environment, hydrophobicity, water content, and pH can influence oxidation selectivity, but a detailed molecular-level understanding of how reactant and product species partition and move within MOF pores remains incomplete.

NU-1000 has shown promising performance in the conversion of CWAs under solar irradiation, particularly for oxidation-based pathways. This behavior is largely attributed to its pyrene-based organic linkers, which contain extended conjugated structures capable of promoting singlet oxygen generation under irradiation, making NU-1000 a desirable catalyst for the oxidation of sulfur mustard and simulants such as 2-chloroethyl ethyl sulfide (CEES).^4^ In addition to its photocatalytic functionality, NU-1000 possesses a hierarchical pore structure composed of microporous and mesoporous regions connected through larger channels, often referred to as c-pores. These different internal environments provide chemically and spatially distinct interfaces: linker-rich confined micropores can promote dispersion-dominated stabilization, whereas mesopore and c-pore regions provide greater free volume and access to hydrated zirconium node sites. This combination of pore environments makes NU-1000 an attractive platform for studying not only catalytic activity, but also the relationship between pore structure, molecular residence, host–guest interactions, and reactant–product transport.^4^

Extensive experimental and computational studies have already established Zr-MOFs as leading candidates for CWA capture and degradation, and NU-1000 is particularly attractive because it combines photoactive pyrene linkers, hydrated Zr nodes, structural robustness, and a hierarchical pore network.^4,11,24–31^ Therefore, the central question addressed here is not whether NU-1000 belongs to the broader class of promising Zr-MOF materials, but how this already promising framework controls the relative residence, transport, and interaction modes of a reactant and its oxidized product. This distinction is important because, in addition to adsorption strength, reactant retention and product mobility represent transport properties that may influence catalytic behavior. It is also worth noting that many studies have explored the role of MOF structures in the degradation of blistering agents; however, comparatively few have examined differences in host–guest interactions between reactant and product species, an important consideration for separations, guest competition, desorption, and reactant–product transport. Limited work has focused on diffusion behavior of blistering agents within heterogeneous pore systems such as NU-1000, where hierarchical pore environments introduce multiple residence and transport regimes. Existing computational and experimental studies often discuss adsorption or diffusion trends in terms of individual structural descriptors, such as surface area, pore volume, or node density, without fully accounting for the combined effects of pore topology, internal interface chemistry, confinement, and molecular polarity on transport. As a result, a molecular-level understanding of how these factors collectively determine self-diffusion, pore preference, and reactant–product differentiation remains incomplete.

In this work, the dual-hosting behavior of NU-1000 toward sulfur mustard (HD) and its partially oxidized product mustard sulfoxide (HDO) is investigated computationally using molecular dynamics (MD) simulations, Monte Carlo adsorption calculations, and density functional theory (DFT) binding-energy calculations. The results reveal that HD and HDO exhibit distinct pore preferences and fundamentally different host–guest interaction modes at comparable molecular loadings. HD preferentially occupies microporous regions, where dispersion interactions and geometric confinement stabilize the molecule and restrict mobility. In contrast, HDO displays a broader spatial distribution, favoring mesopore and c-pore environments that provide greater configurational freedom and access to hydrated zirconium node sites. This divergence in adsorption and residence behavior persists under mixed HD/HDO conditions, indicating that reactant–product differentiation is intrinsic to the NU-1000 pore network rather than an artifact of isolated single-component simulations. By connecting pore occupancy, radial distribution functions, self-diffusion coefficients, and binding-energy calculations, this work identifies NU-1000 as a dual-function molecular host that couples reactant retention with product mobility. These findings provide an interface-level mechanism for selective molecular transport in hierarchical Zr-MOF catalysts and highlight the importance of internal confinement environments in CWA detoxification and related porous-material separation processes.

## 2 Materials and Methods

### 2.1 NU-1000 Surface Topology

A NU-1000 supercell structure containing 12 Zr_6_(μ_3_-O)_8_(μ_3_–OH)_8_ hydrated metal cores with 24 tetratopic 1,3,6,8-tetrakis (*p*-benzoic acid) pyrene (TBAPy^4–^) organic linkers implemented as a periodic heterogeneous catalyst. NU-1000 is composed of Zr_6_-based metal nodes and connected pyrene-based linkers forming a three-dimensional (3D) structure comprising mesopores, micropores, and c-pore channels. The metal node is characterized by six Zr metal sites modeled with alternating terminal −OH and -H_2_O ligands connected directly to the Zr atoms under hydrated conditions.^24^

The NU-1000 framework exhibits a hierarchical pore structure, as shown in Figure 1, with mesopores (∼31 Å diameter) and c-pore channels containing hydrated and catalytically active μ_3_–OH sites, while the micropores (∼8 Å diameter) are bounded by organic linkers and do not contain μ_3_–OH active sites or ligands.^25^ This pore architecture facilitates molecular transport of chemical warfare agents to catalytically active sites. Additionally, the conjugated π-system of the organic linkers promotes the generation of singlet oxygen under photoactivation.^4,26^ The inherent internal hydrophobicity of the NU-1000 structure tends to have high affinity for the nonpolar, hydrophobic species such as HD.^13,27,28^ Due to the strong coordination between the metal nodes and organic linkers, as well as its high structural symmetry, NU-1000 exhibits excellent thermal and chemical stability, even in the presence of humidity.^29–31^ Overall, NU-1000 was selected due to its hierarchical pore structure, accessible active sites, and robust stability, making it an ideal platform for investigating the transport of HD and HDO.^11^

**Figure 1:**
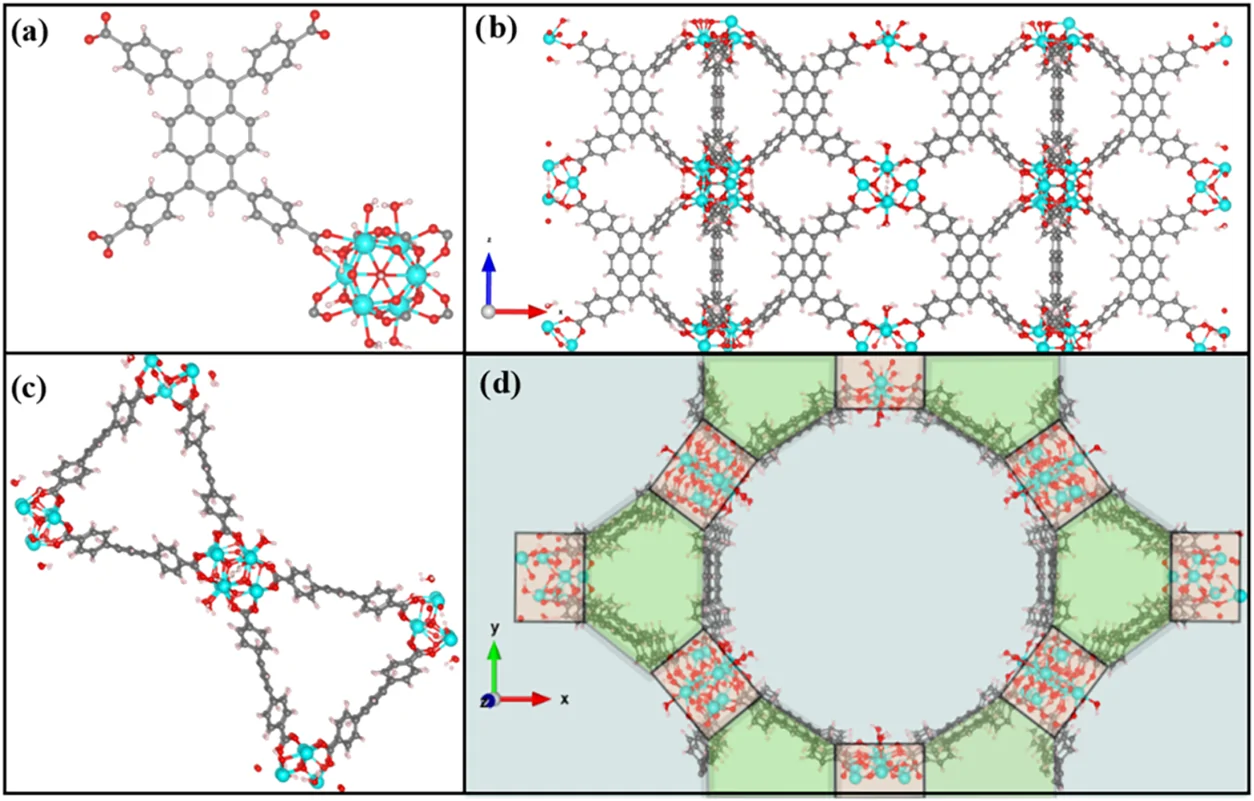
Explanation of topology for NU-1000. (a) Connectivity of metal node (Zr_6_(μ_3_-O)_4_ *(μ_3_ *–** OH)_4_(H_2_O)_4_(OH)_4_) with one organic linker (*tetratopic 1,3,6,8-tetrakis (p-benzoic acid) pyrene (TBAPy*)). There are 8 organic linkers connected to a single node within NU-1000. (b) A perpendicular view to the mesopore to highlight the micropores and c-pore channels. (c) Primitive cell of NU-1000. (d) View to highlight the mesopore (blue) and micropore channels (green), the c-pore channels are perpendicular to this view (tan).

### 2.2 Computational Methods

#### 2.2.1 Molecular Dynamics Calculations

A series of classical MD simulations was performed to gain a clearer understanding of HD (reactant) and HDO (desired oxidized product) dynamics within NU-1000. The MD simulations were executed via the LAMMPS^32^ software (version 23 June 2022) where the mean square displacement (MSD) and radial distribution functions (RDF) were calculated. The nonbonded host–guest interactions were modeled using Lennard-Jones (LJ) with a cutoff radius of 12 Å and Coulomb potentials with a cutoff radius of 10 Å beyond which a particle–particle mesh solver was implemented for long-range electrostatic interactions.^33^ The simulation set contained free guest molecules within a rigid, optimized NU-1000 framework, including pure optimized HD (30 molecules), pure optimized HDO (30 molecules), HD/O_2_ mixture (30/30 molecules), HD/H_2_O_2_ mixture (30/30 molecules), and HD/HDO mixture (30/30 molecules). These low loadings ensured proper host–guest interaction and better represent physically relevant conditions well below pore saturation. The NU-1000 framework was treated as rigid to reduce computational cost and enable longer trajectories focused on HD/HDO host–guest interactions. While framework fluctuations may quantitatively influence diffusion and pore accessibility, particularly within confined pore regions, the rigid representation provides a consistent framework for comparing the relative transport and residence behavior of HD and HDO. NU-1000 is a thermally and chemically stable Zr-based framework, making the use of a fixed crystallographic structure a reasonable approximation for isolating guest–framework interactions over the simulated time scale.^2,30^ The molecules were inserted via PACKMOL^34^ software while partial masses and charges were then assigned through the visual molecular dynamics (VMD)^35^ software package. For each system, ten independent simulations were performed to obtain statistical averages, with uncertainties estimated from the standard deviation across the runs. Each calculation included 50 ns of equilibration under the NVT ensemble conditions followed by 50 ns of production also with the NVT ensemble. Statistical independence between runs was ensured by assigning different random seeds for the initial velocity distributions. The equations of motion were integrated using the velocity-Verlet algorithm with a time step of 1.0 fs, with periodic boundary conditions applied in the x-, y-, and *z*-directions. Structural visualization and analysis were performed using the VESTA software package.^36^

The force field parameters for HD were adopted from a work by Ahady et al., who identified the DREIDING^37^ force field as an appropriate model for describing these interactions.^38^ Although the original DREIDING nonbonded potential uses an exponential-6 form, Ahady et al. converted the nonbonded terms to a Lennard-Jones 12–6 representation to maintain compatibility with the rest of the simulation framework and enable Lorentz–Berthelot cross-interaction mixing. The bonded parameters were implemented using the LAMMPS harmonic bond and angle styles and Fourier dihedral style. Because LAMMPS harmonic potentials omit the conventional 1/2 prefactor, force constants reported in the original harmonic form were scaled accordingly; for example, a DREIDING bond constant of 700 kcal mol^–1^ Å^–2^ corresponds to a LAMMPS input value of 350 kcal mol^–1^ Å^–2^. The HDO force field was constructed using the same DREIDING-based functional form to ensure consistency with HD, with shared bonded and nonbonded parameters retained where applicable and additional S–O terms were assigned for the oxidized sulfur center. Partial charges for HDO and HD were determined with the DMol^3^ software^39^ within BIOVIA Materials Studio^40^ commercial software, where both Mulliken and Hirshfeld charges were compared to determine the effects of charge transfer from the introduction of oxidation. Mulliken charge analysis is simple and readily available in DMol;^3^ however, known limitations include significant variations in atomic charges with small changes in the basis set and a tendency to overestimate covalent character.^41^ Often hydrogen atoms near halogens lead to artificially enhanced charges which are highly dependent on the chosen functional affecting larger atoms.^42^ The comparison between CHelp method^43^ within Gaussian 09 ^44^ from reference^38^ and the Hirshfeld population analysis within DMol^3^ proved similar pattern of preferential location and diffusion patterns for HD within NU-1000. Therefore, continued use of DMol^3^ Hirshfeld population analysis for HDO is used to analyze diffusion of the oxidized product within NU-1000. Force field parameters for NU-1000 were collected by Oliver et al.^2^ where it was determined that a combination of DREIDING, UFF^45^ and TraPPE^46–48^ potentials was ideal for describing host–guest interactions. Previous works include this combination approach with DREIDING and UFF while Wardzala et al. suggested the use of TraPPE force field parameters for the μ_3_–OH groups to improve hydrogen bonding networks and adsorbate interactions.^49^ TraPPE force field parameters were also used for the O_2_ molecules. Hydrogen peroxide (H_2_O_2_) was described using parameters reported by Orabi et al. where a two-site model consisting of oxygen and hydrogen atoms was employed.^50,51^ This model has been shown to reproduce the dipole moment and intermolecular interactions of H_2_O_2_ in previous studies. Partial charges of −0.41 and +0.41 were assigned to the oxygen and hydrogen atoms, respectively, to reproduce the molecular dipole. Cross interactions were handled using Lorentz–Berthelot mixing rules.

The time dependent calculations carried out by LAMMPS in this work include radial distribution function and mean square displacement. The self-diffusion coefficients for guest molecules within the NU-1000 framework were estimated with calculations of MSD from Einstein relation where the slope of the MSD vs time is proportional to the diffusion coefficient shown in eq 1

1 $$D=\frac{1}{2d}\lim_{t\to \infty }\frac{\left\langle {\left[z\left(t\right)-z\left(0\right)\right]}^{2}\right\rangle }{t^{\alpha }}$$

where *D* is the self-diffusivity, d represents the dimensionality of motion which is three as this is a three-dimensional system, *t* is simulation time, *z*(*t*) is particle position at a given time and α represents the restriction on the motion present during interactions. The calculated diffusion exponent of α = 0.981 approaches unity, supporting the assumption of approximately Fickian long-time diffusion. The initial position *z*(0) is considered the first step of the production calculation as this is an observable quantity.

RDFs were calculated between guest atoms and specific predetermined framework sites (μ_3_–OH, Zr–OH, Zr–H_2_O) with a maximum radial cutoff of 10 Å to quantify site-specific interactions. The calculation was employed within the LAMMPS script following eq 2

2 $$g\;\left(r\right)=\frac{\left\langle \rho \left(r\right)\right\rangle }{\rho }$$

where ρ represents the bulk density of particles and ρ­(*r*) represents the local density of specific guest particles from a particular site at a particular distance (*r*). The RDF and MSD values were calculated for all sets of calculations separately to ensure robust, accurate calculations.

#### 2.2.2 Monte Carlo Calculations

Adsorption loading calculations were performed using the Sorption tool at 300 K, with fugacity values ranging from 0.0001 to 25 kPa. The COMPASS III^52^ was employed, and the Metropolis Monte Carlo method was applied.^53^ Nonbonded interactions were treated with a 12.5 Å cutoff. Each data point at a fixed fugacity was sampled in the grand-canonical (μVT) ensemble using Monte Carlo insertion, deletion, translation, and rotation moves with relative probabilities of 2:1:1:1

#### 2.2.3 Density Functional Theory Calculations

The binding energies of mustard gas within the NU-1000 framework were determined using DFT calculations as implemented in the *Vienna Ab initio Simulation Package* (VASP 6.4.1).^54–56^ The projector augmented-wave (PAW) method was used to describe core–electron interactions, in combination with the Perdew–Burke–Ernzerhof (PBE)^57^ exchange–correlation functional. A plane-wave energy cutoff of 520 eV was applied for all calculations. Electronic convergence was set to 1 × 10^–6^ eV, with a maximum of 300 self-consistent field (SCF) iterations. All calculations were performed at the Γ-point, appropriate for the large unit cell of the MOF system.^58^ Dispersion interactions were accounted for using the DFT-D3 method with Becke–Johnson damping.^59,60^ Dispersion corrections are essential for accurately describing host – guest interactions in porous materials. Geometry optimizations were carried out using the conjugate gradient algorithm, with a force convergence criterion of 0.05 eV/Å and a maximum of 400 ionic steps. The lattice parameters were held fixed during optimization, while atomic positions were allowed to relax. The positions of the guest molecules within the host were determined through RDF, trajectory analysis, and manual probing of at least three different configurations within the range of the predetermined RDF values. This analysis was constructed as validation of the calculated resident time and RDF calculations performed within the molecular dynamics simulations and not for probing all possible sites and configurations that could occur for each of the guest molecules within NU-1000.

After the geometry optimization of the HD and HDO in NU-1000, single point energies of the combined systems as well as the isolated pore and substrate systems were calculated. From here, the binding energy was calculated according to

$$E_{bind}=E_{HD+MOF}-\left(E_{MOF}+E_{HD}\right)$$

With a similar expression for HDO. where *E*_HD+MOF_ corresponds to the total energy of the optimized adsorbed system, and *E*_MOF_ and *E*_HD_ are the energies of the isolated framework and molecule, respectively, computed using single-point calculations at the same level of theory. Negative binding energies indicate favorable adsorption.

We also want to note that no experimental handling of sulfur mustard or related chemical warfare agents was performed in this work. All results and discussions below are based on molecular models and computational simulations.

## 3 Results & Discussion

### 3.1 Adsorption Loading as a Boundary Condition for Low-Loading Transport

Grand-canonical Monte Carlo simulations indicate that HD and HDO exhibit comparable adsorption behavior within NU-1000, with saturated loadings of 395 and 378 molecules per unit cell, respectively. This similarity reflects the comparable size and overall molecular structure of the two species. For the following MD simulations, a low loading of 30 molecules was selected to avoid complications arising from pore saturation or significant guest–guest interactions. Such conditions are representative of dilute environments where transport and adsorption behavior are governed primarily by interactions with the framework.

The results presented in Figure 2 are an indication for maximum loading for MD specific calculations. The experimental nature of interactions between CEES/HD and the NU-1000 framework include weak polar interactions between the guest sulfur atoms and zirconium metal sites while evidence of hydrogen interactions between S/Cl atoms in CEES/HD occur with the hydroxyl and hydrated metal sites.^31,61^ Various van der Waals forces between the CWA and carbon linkers has also been shown to occur to add continued stability while influencing adsorption and desorption energy.^61–63^ Alkanes are often used to indicate similar types of van der Waals interactions that could be present in the linear HD molecule with NU-1000, however there are significant differences in the host–guest interactions. More robust adsorption isotherms shown in a work presented by Vargas L. et al. regarding alkanes in NU-1000 indicates preferential micropore and microchannel filling. As the concentration of the alkanes increases, the mesopores begin to fill once the microchannels reach full capacity.^64^ Further studies show alkene/alkane loading in MOFs containing hierarchical pore structure observes similar trends with a magnitude correlating to the distinct pore patterns.^63,65^

**Figure 2:**
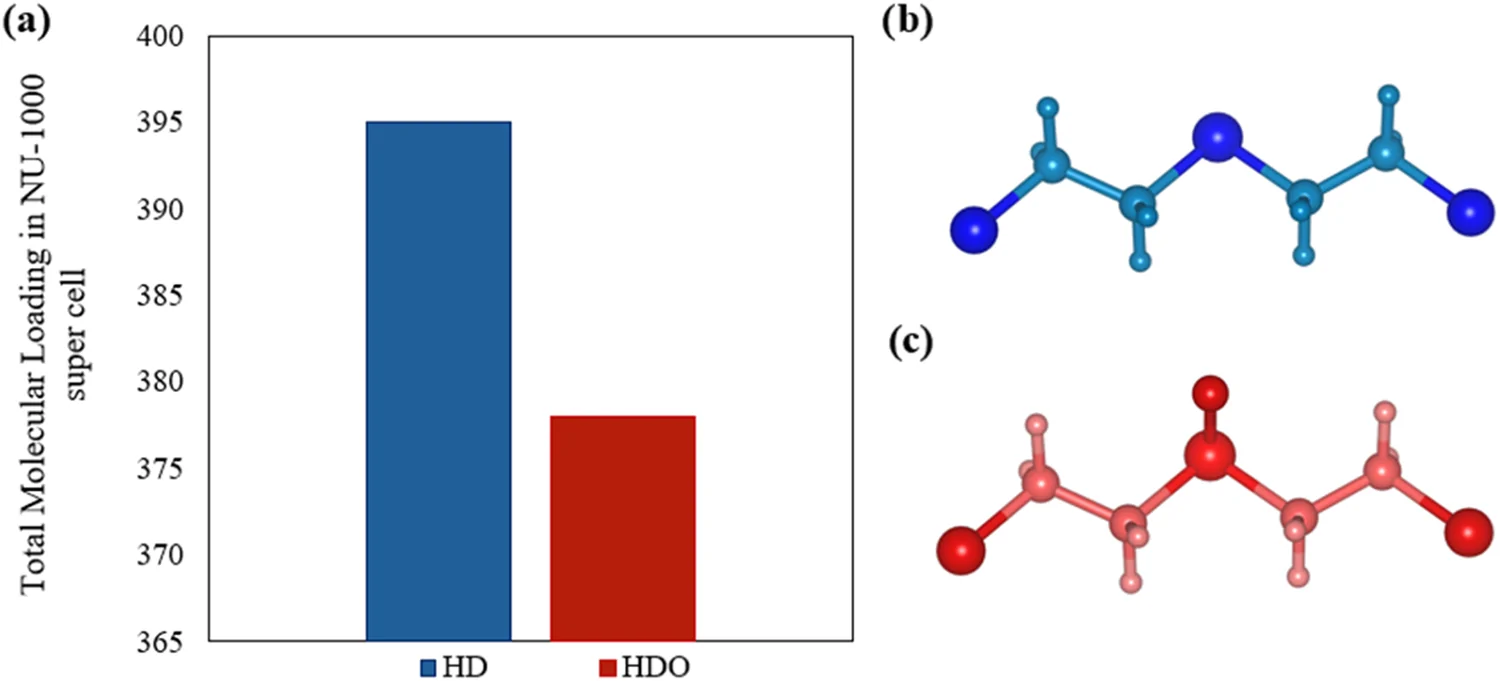
Sorption calculation carried out in Materials Studio for the low loading justification. (a) Final equilibrated guest distribution within the NU-1000 supercell. (b) Optimized molecular structure of sulfur mustard (HD), represented in blue throughout this work. (c) Optimized molecular structure of mustard sulfoxide (HDO), represented in red throughout this work.

Experimental adsorption research by Son et al. observed the comparative adsorption of CEES, a mustard gas simulant used in experiments as it proves to be a safer chemical with similar structural and chemical properties, within a variety of MOFs.^28^ Conclusively, adsorption of CEES in MOFs is enhanced within smaller pore environments with large surface areas where the highest loadings were found within NU-1000 (21.3 mol/kg) and NU-1200 (22.6 mol/kg) indicating surface area and node connectivity plays a considerable role in the adsorption of CWA HD. The calculated loading of HD in NU-1000 within MC indicated a slightly lower loading of 15.1 mol/kg possibly attributed to the larger structure of HD compared to CEES or changes in interactions. While hydrogen interactions play a key role in the attraction between CWA and MOF-host, evidence of significantly lower loading of CEES/HD in hydrophilic MOF environments indicated a stronger affinity for the acid-site hydrogen bonding interactions with water rather than that of HD.^28^ Interestingly, the CEES adsorption in NU-1000 was shown to exhibit higher loadings in humid conditions compared to dry conditions, vastly differing from the humid/dry adsorption patterns observed from other, more hydrophilic MOFs. Discrepancies among the adsorption patterns of CEES/HD in a variety of MOFs suggest multitiered factors that cannot be limited to a single topological descriptor. Pore topology, surface area, linker connectivity, and linker functionality all play a role in the uptake and diffusion patterns exhibited by CWA molecules while the influence of each structural quality still remains uncertain.

A direct correlation between pore volume, surface area, and CEES loading is undeniable though not a determining factor. Comparatively MOF-525 has a larger surface area and pore volume than MOF-808 but significantly lower loading indicating perhaps node connectivity and terminal −OH, −H_2_O molecules play a larger role. Preferential pore loading of variants of HD in NU-1000 indicates micropore loading at low fugacity with pore filling mechanism and preferences for confinement interactions. At higher loading values mesopore channels begin to fill until a max capacity is reached dependent on the type of MOF, pore structure, available metal sites, and ligand types. The results within Figure 2 are used to demonstrate maximum loading at high fugacity for time dependent calculations and not necessarily pore filling mechanisms. These are instead justified through literature research.^66^

### 3.2 Pore-Selective Dual Hosting of HD and HDO

Within the LAMMPS calculations, low loading of HD and HDO were individually analyzed to determine the differences between preferential pore locations to better understand reactant/product diffusion patterns for insight into possible separation pathways. These findings were then compared to a mixed product/reactant simulation for validation of preferential pore locations under a combined loading environment. Low loading molecular dynamics simulations reveal pronounced differences in pore occupancy between HD and HDO within NU-1000 (Figure 3a). HD exhibits a strong preference for the microporous region, spending 79.9% of the trajectory confined within the micropore, with only minor sampling of the mesopore (2.7%) and c-pore (17.4%). In contrast, HDO displays a markedly broader spatial distribution: 44.8% mesopore occupancy, 33.8% c-pore sampling, and only 21.4% confinement within the micropore.

**Figure 3:**
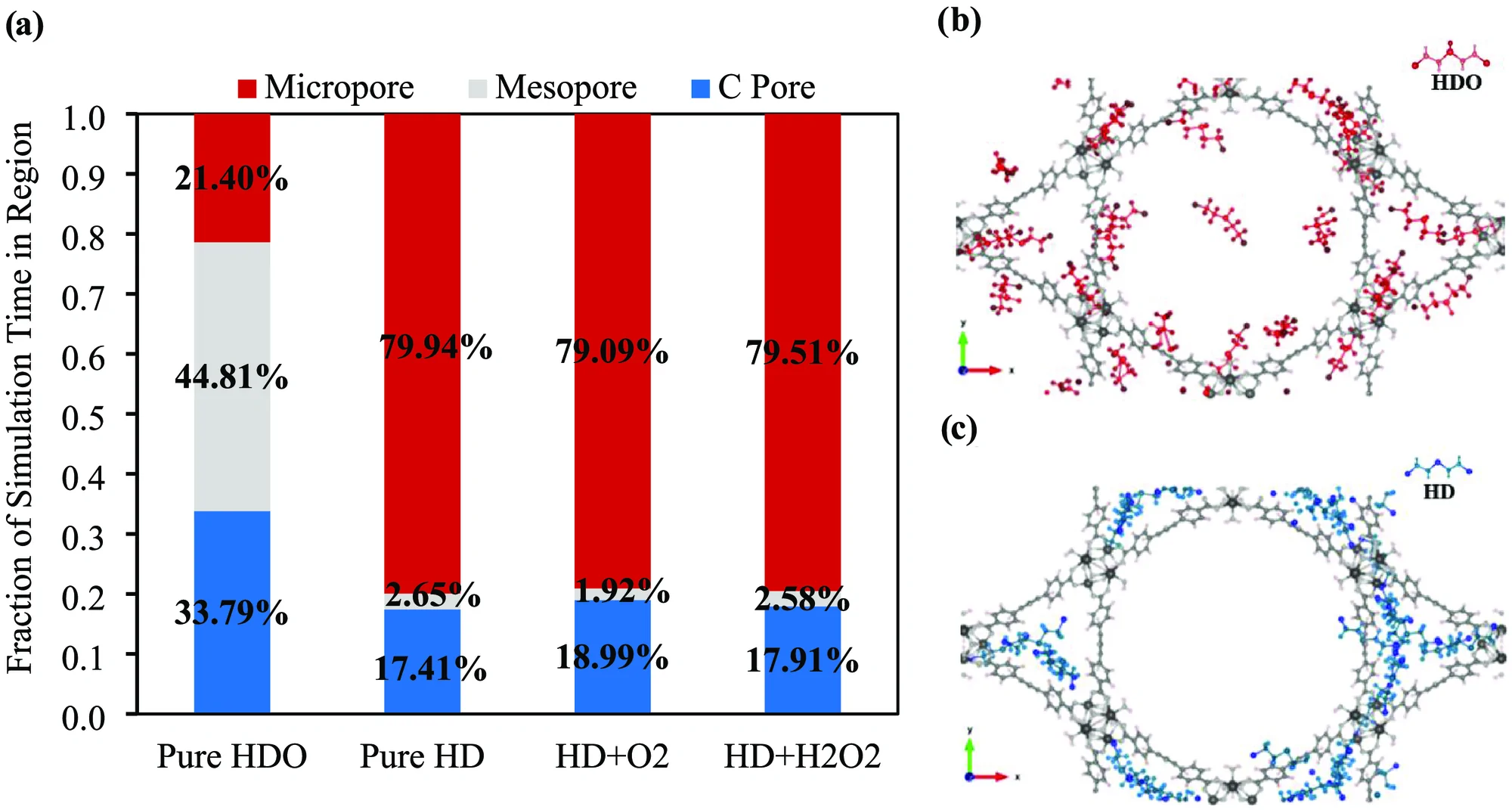
Distribution analysis of HD and HDO in NU-1000. (a) Fraction of simulation time spent in mesopore, micropore, and c-pore for pure HDO, pure HD, HD (HD+O_2_), and HD (HD+H_2_O_2_). (b) Representative trajectory density of HDO showing broad sampling of mesoporous and c-pore environments. (c) Representative trajectory density of HD demonstrating strong localization within the micropore region. Pore-residence fractions exhibited minimal variability across the ten independent MD trajectories.

The trajectory snapshots (Figure 3b,c) visually reinforce these quantitative differences. HD is highly localized along the micropore channel walls, whereas HDO exhibits diffuse sampling throughout the mesoporous cavity and c-pore windows. This divergence in pore preference is mechanistically significant. The micropore region of NU-1000 lacks μ_3_–OH and terminal Zr–OH/Zr–H_2_O sites, indicating that HD confinement is not driven by specific coordination interactions. Instead, its strong localization likely arises from confinement stabilization and dispersion-dominated interactions within the narrow microporous environment.

In contrast, the broader mesopore and c-pore sampling of HDO suggests that polarity and hydrogen-bonding capability influence its spatial distribution. The additional oxygen atom in HDO increases its dipole moment relative to HD, enhancing electrostatic and hydrogen-bonding interactions with hydrated zirconium nodes located in the mesopore and c-pore domains. This interaction propensity likely reduces the thermodynamic driving force for strict micropore confinement and instead prefers site specific configurations that limit other guest species within the vicinity to interact simultaneously. As a result, HDO preferentially occupies larger pore regions that speculatively provide sufficient configurational freedom to accommodate these interactions.

Analysis of HD and HDO transport within NU-1000 was further analyzed using a mixed system containing 30 HD and 30 HDO molecules. This mixed system analysis was performed to determine whether the pore-selective behavior observed in isolated simulations persists under competitive coadsorption conditions. As shown in Figure 4, the coexistence of reactant and product species maintains the same preferential pore occupancy trends observed in the individual systems. HDO continues to favor mesopore and c-pore environments due to orientation-dependent interactions between the sulfoxide oxygen and hydrated zirconium node sites, which sterically limit efficient confinement within the micropore. In contrast, HD retains strong localization within the microporous regions, where its linear and relatively nonpolar structure allows favorable confinement and dispersion-driven interactions with the framework. These results demonstrate that the spatial separation behavior of HD and HDO is preserved even in mixed reactant – product environments.

**Figure 4:**
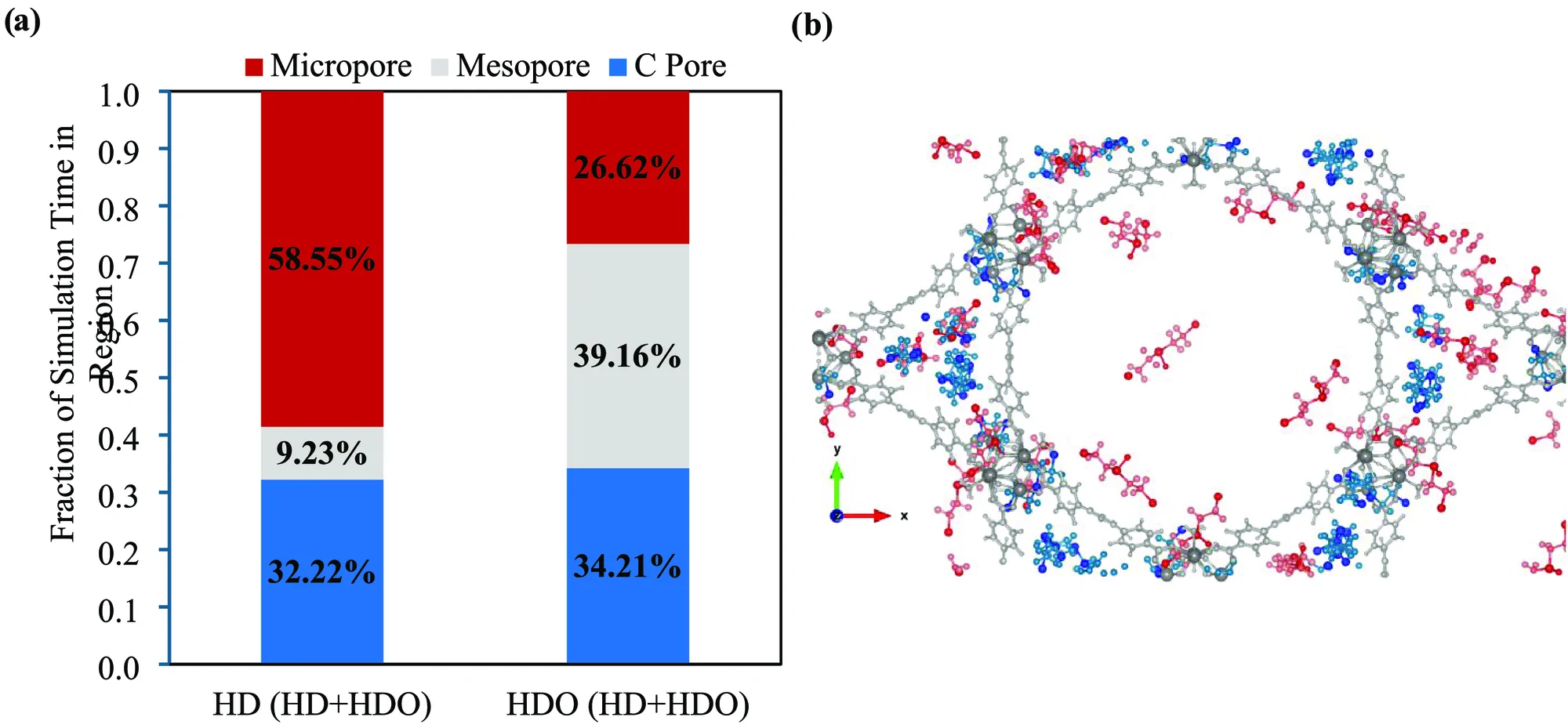
Mixed reactant–product system containing 30 HD and 30 HDO molecules within NU-1000. (a) Fractional pore occupancy of HD and HDO within the mixed system, illustrating that the distinct pore preferences of reactant and product species are maintained under coadsorption conditions. (b) Trajectory snapshot of equilibrated HD and HDO mixture within the hierarchical pore structure of NU-1000. Pore-residence fractions exhibited minimal variability across the ten independent MD trajectories.

These contrasting residence profiles may have important implications for transport and separation. Micropore localization of HD could lead to longer residence times and diffusion-limited desorption, whereas the mesopore-accessible HDO may experience facilitated transport and more rapid release. Such behavior suggests that inherent pore-dependent stabilization differences can contribute to emergent selectivity between reactant and product species in NU-1000 based catalytic systems

### 3.3 Internal Interface-Specific Host–Guest Interactions

Alternative approaches for determining site specific interactions for time dependent calculations include RDF analysis, where the first peak of any RDF calculation is an indication of the preferential location of guest molecules to specific framework sites. While active sites are typically a strong indication of chemisorbed, catalytic reaction, the active sites associated with the capture of HD and alkanes alike should be considered more like stabilization sites, where the adsorbate is preferentially located near and energetically minimized within the vicinity of such host sites. Figure 5c includes a trajectory snapshot of HD within micropores and a c-pore where the configurations of the individual HD molecules show the “stretching” configurations across organic linkers from one node to another. Perhaps, for this type of molecule, the most preferential node/ligand ratio for a MOF would be one that allows for adequate interactions between known active sites with each of the chlorine atoms while the entirety of the molecule is energetically stabilized with organic linker interactions through van der Waals forces.

**Figure 5:**
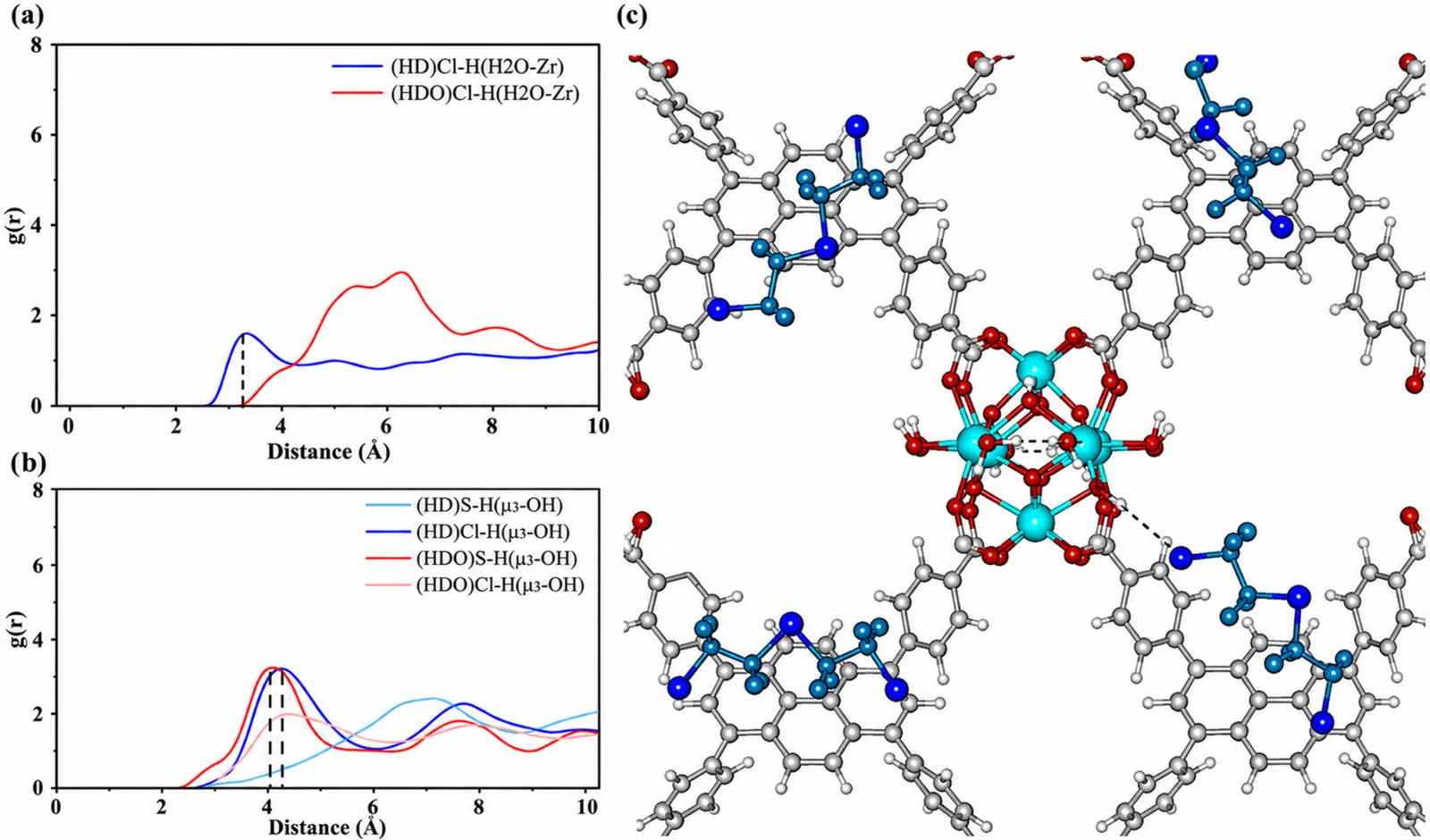
Radial distribution plots of HD and HDO within NU-1000 and representative configuration of HD from MD simulations. (a) RDF of HD and HDO chlorine atom interactions with hydrated Zr–H_2_O sites on the metal node. (b) RDF of HD sulfur and chlorine atoms and HDO sulfur and chlorine atoms interacting with the μ_3_–OH sites on the metal node. (c) Representative configuration of HD preferentially occupying the micropore and c-pore regions, highlighting favorable interactions with both the organic linkers and metal node sites. The dotted line indicates a favorable interaction between an HD chlorine atom and a μ_3_–OH site. RDF uncertainties across ten independent MD trajectories were minimal, with an average standard deviation of σ < 0.005.

The chlorine −hydrogen interactions associated with this stretching configuration are demonstrated with the RDF plot in Figure 5a, where the first peak of HD chlorine average density is within ∼3.2 A, a similarly determined location for minimum binding energy for HD within c-pore/micropore regions as shown in Figure 8. These clues allow for the validation of experimental conclusions indicating chlorine hydrogen interactions stabilize HD confinement within NU-1000.^62^ Interestingly, the presence of humidity has shown to increase the adsorption of HD in NU-1000,^28^ supported by the MD analysis studies presented which indicate the HD interacts favorably with the hydrated sites as means of weak halogen–hydrogen interactions.

Comparison of HD and HDO sulfur atoms with hydrogen atoms at the μ_3_–OH sites shown in Figure 5b reveals the preferential configuration differences between the two adsorbed molecules. Clear peaks for HD mostly include chlorine interactions at μ_3_–OH sites, indicative of preferential halogen interactions with hydrogen within the pore network. Importantly, although the center of mass of HD is predominantly assigned to the micropore during the pore-residence analysis, the terminal Cl atoms can extend toward μ_3_–OH sites associated with the adjacent c-pore environment. Thus, the HD–Cl···H­(μ_3_–OH) peak does not indicate the presence of μ_3_–OH sites within the micropore, but instead reflects an interfacial configuration in which HD stretches between the micropore and neighboring c-pore/node environment. Some HD transport through the c-pore is also observed and may provide a smaller contribution to these interactions. This configuration is consistent with the elongated structure of HD and its preferential interactions across organic linkers connecting the metal nodes. In contrast, a heightened peak for HDO between the central sulfur atom and μ_3_–OH sites shown within Figure 6 emphasizes to the differences in configurations between product and reactant, where HDO tends to favor electronegative oxygen interactions with the metal node, limiting the ability for effective pore filling for HDO within more constrained environments. The oxygen within the oxidized HDO species introduces a polarity that is otherwise absent within the linear HD structure. HDO provides a less hydrophobic structure that has more direct interactions between the HD=O group and the hydrated -H_2_O ligand on the Zr atoms.^31,67^ This configuration limits the ability for HDO to coexist with other HDO molecules around the active sites, pushing HDO to the mesopore and increasing diffusion coefficients in the attempts to satisfy the possibly entropically limiting preferential adsorbate–host interactions. This is further highlighted in the binding energy calculations where apparent preferential configurations limit linker and HDO interactions but increase S–O and metal node interactions.

**Figure 6:**
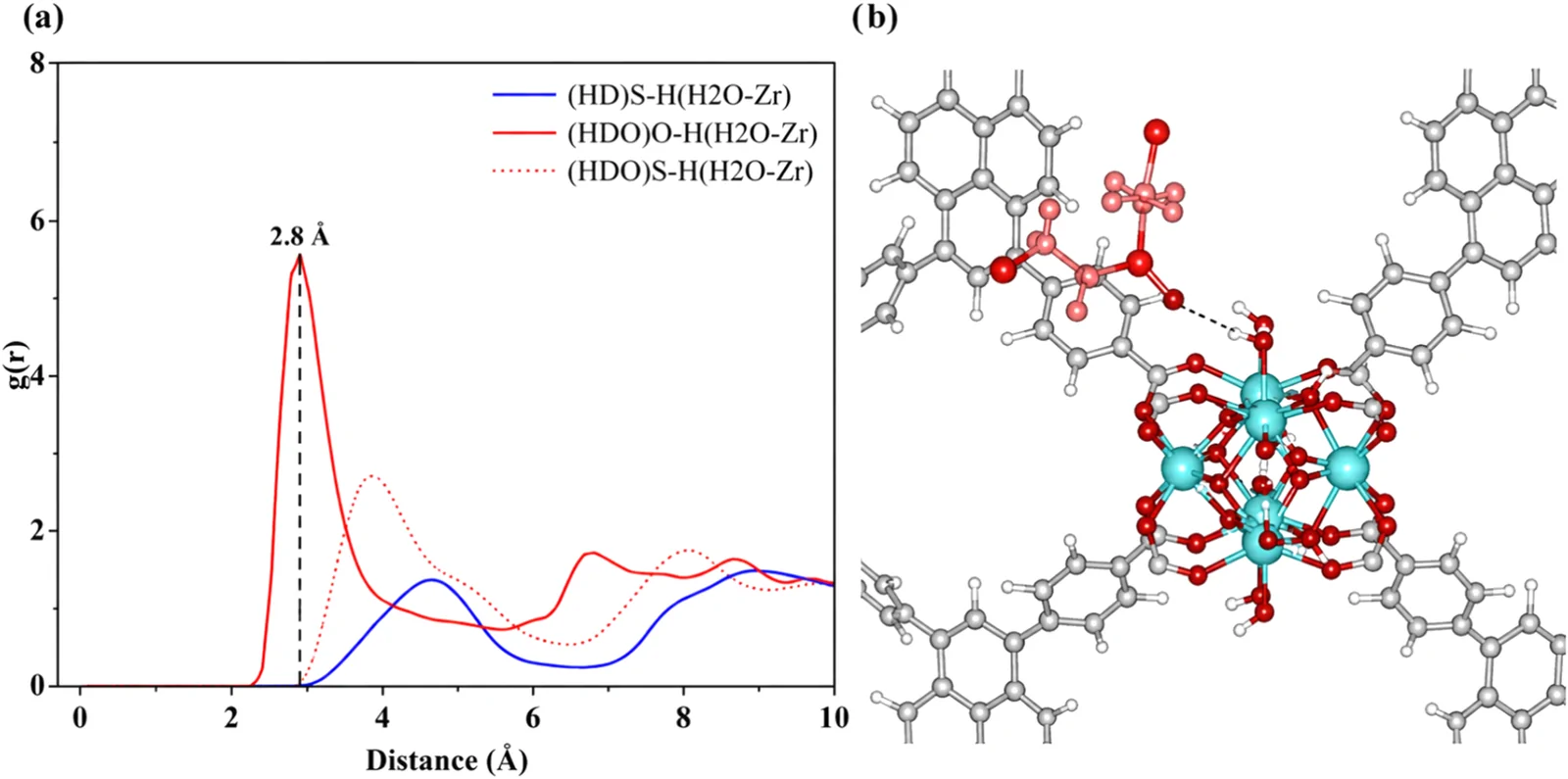
(a) RDF calculation comparing central atoms sulfur in HD and sulfur and oxygen in HDO compared to the hydrogen atoms on hydrated Zr sites. (b) A trajectory snapshot of HDO oxygen interactions with the hydrated Zr-metal sites. RDF uncertainties across ten independent MD trajectories were minimal, with an average standard deviation of σ < 0.005.

These differences in site-specific interactions provide a molecular-level explanation for the observed divergence in pore preference and diffusion behavior. HD stabilization through distributed interactions promotes confinement and reduced mobility, whereas HDO stabilization through localized, orientation-dependent interactions facilitates broader spatial sampling and enhanced diffusion.

### 3.4 Transport Asymmetry between Confined Reactant and Mobile Product

The self-diffusion rates of HD, HDO, and HD in the presence of O_2_ within NU-1000 were quantified through molecular dynamics simulations, shown in Figure 7. To maintain statistical relevance, 10 independent trajectories were evaluated for each unique system. The results indicate that HDO possesses self-diffusion values ranking more than an order of magnitude greater than those of HD or HD+O_2_ systems. Specifically, HDO exhibits a self-diffusion rate of approximately 10.6 × 10^–10^ m^2^/s, while HD and HD+O_2_ show much lower diffusion rates of ∼0.5 × 10^–10^ m^2^/s and ∼0.2 × 10^–10^ m^2^/s, respectively. These differences highlight the pronounced effect of molecular size and framework interactions on mobility within the MOF. The HDO trajectory reflects a wider distribution of accessible pathways, consistent with its preferential occupancy of mesopore and c-pore regions, which are less sterically constrained compared to the micropores favored by HD. Furthermore, HD exhibits stronger interactions with the porous framework as described by the binding energy relationships. Between van der Waals interactions, the chlorine – hydrogen interactions, and the sulfur proximity to hydrated zirconium environments, HD maintains a small level of motion mainly restricted to traveling between micropores through c-pore channels. Validation of true diffusion and not purely contained motion was validated through the trajectory which showed while HD tends to remain in micropores, movement through the c-pore is observed as shown in the provided trajectory snapshot (Figure 7b).

**Figure 7:**
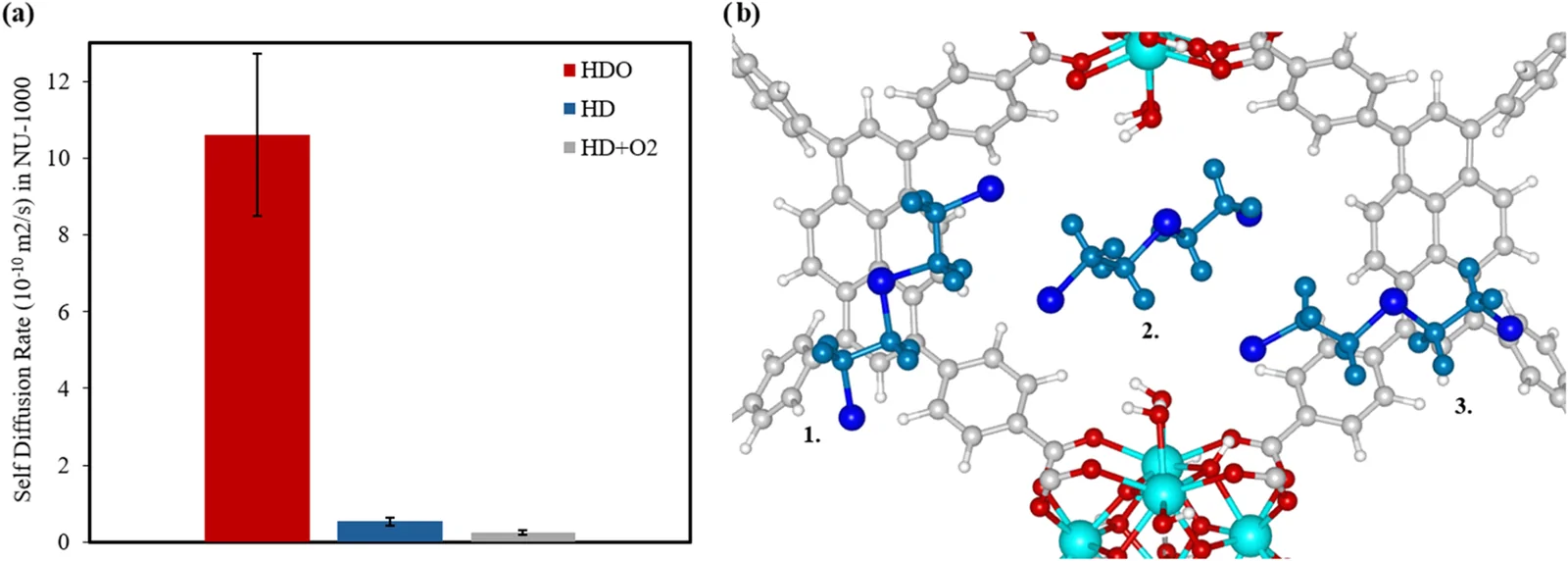
(a) Self-diffusion patterns captured within LAMMPS from MSD calculations. HDO (Red) *D*_self_ = 10.6 ± 2.11 × 10^–10^ m^2^/s; HD (Blue) *D*_self_ = 0.5 ± 0.09 × 10^–10^ m^2^/s; HD+O_2_ (Gray) *D*_self_ = 0.2 ± 0.06 × 10^–10^ m^2^/s. (b) Trajectory snapshots from HD moving through c-pore between micropores.

Although most diffusion coefficient values are limited to computational works, particularly for the diffusion of CWAs within MOFs, self-diffusion coefficients of simulants such as alkanes have been rigorously studied. Self-diffusion coefficient values for *n*-hexane within NU-1000 have been shown to exhibit values around ∼10^–10^–10^–9^ m^2^/s, which varies according to loading and temperature.^68–70^ With this in mind, halogen bonding plays a large role in the surface interactions of HD within NU-1000 so decreased diffusion values for HD and HDO are expected. To further evaluate loading effects, HDO self-diffusion was calculated over a range of 5–25 molecules per NU-1000 unit cell (Figure S1), with the corresponding pore distributions shown in Figure S2. Increasing HDO loading resulted in an approximately order-of-magnitude increase in the calculated self-diffusion coefficient. These results indicate that pore filling can significantly influence HDO transport and distribution within NU-1000. In contrast, at loadings below 30 molecules, HD remained predominantly localized within the confined micropore and c-pore environments with comparatively smaller changes in its diffusion behavior. These loading-dependent simulations demonstrate that guest concentration, particularly for HDO, can influence the quantitative transport behavior observed within NU-1000. Within the binding energy calculations, we find that HD has a more preferable interaction with the organic linkers as compared to HDO which allows for more linear, hydrophobic HD molecules to effectively pack within the c-pore and micropore channels, stretching themselves between metal node sites.

The stark contrast in diffusion behavior between HDO and HD with similar loading values emphasizes the role of NU-1000 in promoting selective transport within its hierarchical pore network. As HD is largely confined to micropores, restricting its mobility, HDO benefits from the more open mesopore channels, facilitating rapid transport. The presence of O_2_ further reduces the mobility of HD, which is attributed primarily to decreased accessible free volume and increased molecular crowding within the pore network. No substantial change in HD conformation was observed between the HD and HD+O_2_ trajectories, suggesting that the reduced diffusion instead arises from hindered dynamics and potentially additional weak HD–O_2_ intermolecular interactions. These findings suggest that the spatial separation of reactants and products not only influence adsorption but also significantly impacts diffusion kinetics. The simultaneous retention of HD and greater mobility of HDO demonstrates a reactant–product transport asymmetry that could be relevant to product removal during catalytic oxidation; however, the present simulations do not explicitly model the oxidation reaction or catalytic turnover.

The rates determined within Figure 7 convey the self-diffusion values for the MSD calculation over the entire production trajectory. A formalized method by Savin and Doyle^70^ referred to as the finite subvolumes method^64^ determines the diffusion coefficient of guest particles in porous environments according to pore size. In a work by Vargas L. et al., this method was invoked for the self-diffusion rates of alkenes in NU-1000 where the subvolumes were the mesopores and micropores. They found that diffusion of longer chain alkenes such as butane exhibit an order of magnitude lower self-diffusion coefficient in the micropore compared to the mesopore.^64^ Similarly, the stark differences in diffusion coefficients and preferential pore location for HD and HDO within NU-1000 found in Figures 7 and[Fig fig3] show that the determined self-diffusion of HDO might be significantly larger than HD because of the mesopore preferences for loadings of 30 molecules. This is subject to change according to the loading chosen. The adsorption of HDO in the smaller pore environments shows favorable interactions for HDO in the micropore and c-pore, however the loading amount for the micropore might not be as great as HD because of steric hindrance and the affinity for favorable interactions between HD and the organic linkers. Pore preferences are likely a combination of guest – host interactions, adsorption properties, diffusion path preferences, and confinement constraints. HDO’s preference for larger pore volumes at a low loading such as exhibited in the MD calculations exhibits possibilities for adsorbate selectivity and the ability for product–reactant separation within a single heterogeneous pore environment possibly without the need for additional separation methods. Diffusion patterns within MOFs cannot be limited to a single topological factor but rather final observations include the need for systematic studies of mass transfer coefficients within hierarchical pore structures where increased loading leads to increased self-diffusion coefficients as well as increased loading leads to changes in preferential pore distributions.

### 3.5 Binding-Energy Support for Pore-Dependent Stabilization

Through the combination of hydrogen interactions with the Zr–OH and –H_2_O ligands and the μ_3_–OH sites on the metal node with the Cl^1–^ and S^2–^ atoms within HD, a negative/favorable adsorption energy value was evaluated through the VASP DFT calculations. A series of calculations including geometry optimization using the PAW–PBE functional, followed by single point energy calculations involving each individual component allows for comparable binding energy calculations within different porous regions of the NU-1000 structure to aid in determining energetically favorable adsorption locations for HD and oxidized product HDO.

The binding energies for mustard gas exhibit a preference for the c-pore with a binding energy of −12.5 kcal/mol. Overall, between HD and HDO, this is the strongest binding energy interaction with NU-1000 among the sampled locations and configurations. This is likely due to the confinement effect that exhibits van der Waals interactions between the hydrophobic pores of NU-1000 and the linear HD structure. Similarly, for the micropore interactions for HD, a favorable binding energy was determined to be similar to that within the c-pore channel with around −12.0 kcal/mol average binding energy depending on the configuration or location in the pore. The binding energy for HD within the mesopore was significantly lower than the binding energies expressed in the confined spaces of the micropore and c-pore channels with a binding energy value of less than −1.0 kcal/mol indicating preferential confinement in the micropore and c-pore. It is important to note that these binding energy calculations isolate host–guest interactions and do not account for guest–guest interactions or dynamic effects, which are captured in the time dependent molecular dynamics simulations. Therefore, binding energies provide a thermodynamic perspective on preferred interaction sites, whereas MD simulations reflect the combined influence of enthalpy, entropy, and steric accessibility on pore occupancy and transport.

For catalytic oxidation processes, the balance between reactant adsorption and product desorption can be an important consideration. The predominantly physisorptive interactions observed here indicate that HD and HDO remain favorably associated with NU-1000 while retaining the ability to undergo molecular transport within the pore network. In particular, the greater mobility of HDO relative to HD suggests that the oxidized product may be more readily transported away from confined regions. Such reactant–product transport asymmetry could be relevant to catalytic systems where product removal is desirable; however, catalytic reaction kinetics, selectivity, and turnover are not explicitly evaluated in the present simulations (Figure 8).

**Figure 8:**
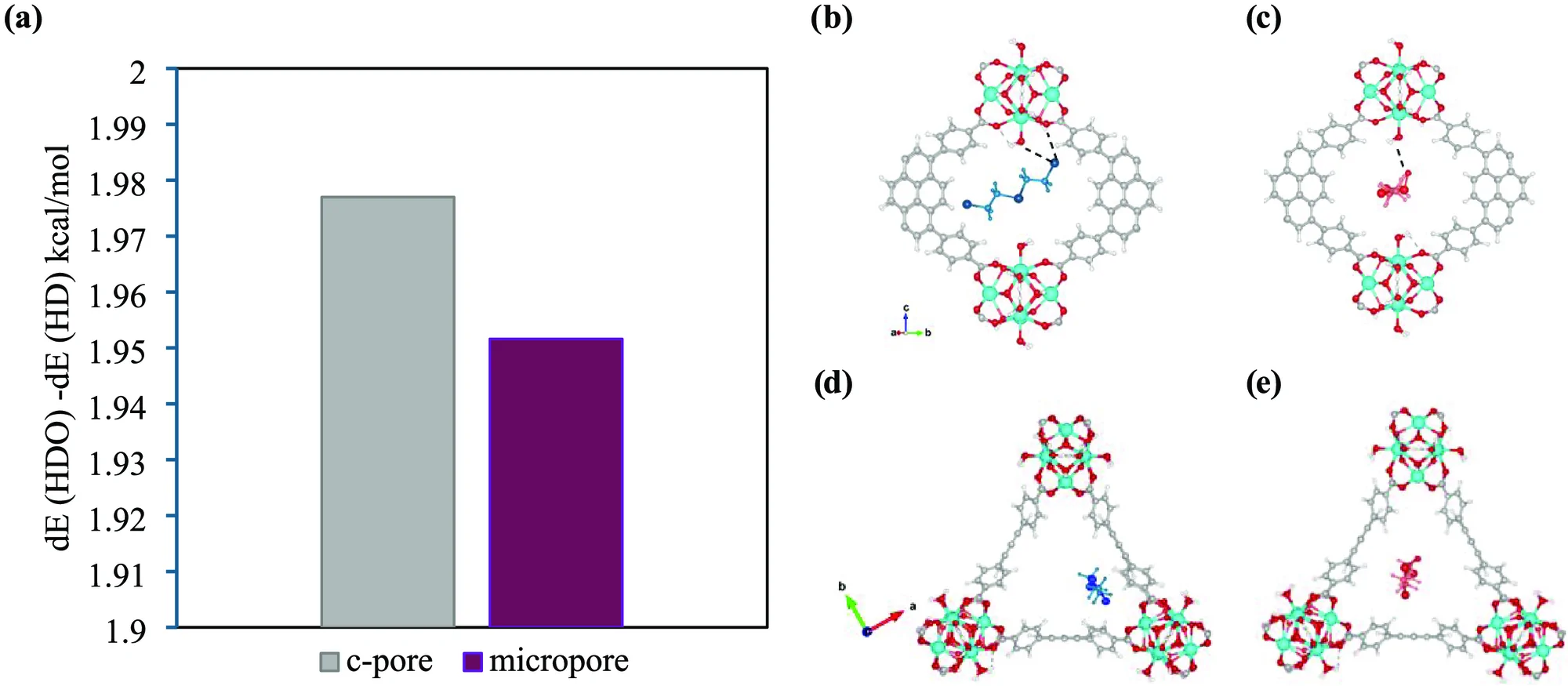
(a) Binding energy comparison for HD and HDO in the c-pore and the micropore where a negative dE (HDO)- dE (HD) represents more favorable HDO binding and a positive dE (HDO) - dE (HD) represents a more favorable HD binding. (b, c) HD (blue) and HDO (red) lowest energy configurations within the c-pores. (d, e) HD and HDO lowest energy configurations within the micropores.

HDO exhibits a similar trend with the highest binding energy expressed in the c-pore, closely followed by the micropore and then last the mesopore. There are significant indications of van der Waals influence on the confinement effect for both HD and HDO in NU-1000. Although similar trends in binding energies between the product and reactant are expressed, systematic studies of HD and HDO pore locations give way to an understanding that HD interacts more preferentially with the NU-1000 organic linkers while HDO has more significant interactions with the metal node and node ligands. Figure 9 highlights some of the mesopore specific studies within the series of calculations. Initially HD and HDO were placed in similar locations over the organic linkers in the mesopore as indicated by Figure 9c,d. Conclusively HD indicated a stronger preferential binding energy for the location over the organic linkers, which is also shown and evaluated in Figure 5, when compared to HDO. However, HDO indicates a stronger preferential binding energy over the metal node when compared to HDO over the organic linker as graphed in Figure 9 where a more negative value is expressed for HDO over node. Although the binding energy for the mesopore was lower than the expressed binding energies in the c-pore and micropore, HDO–HDO interactions were not evaluated, and preferential pore loading was not indicated through simple binding energy calculations.

**Figure 9:**
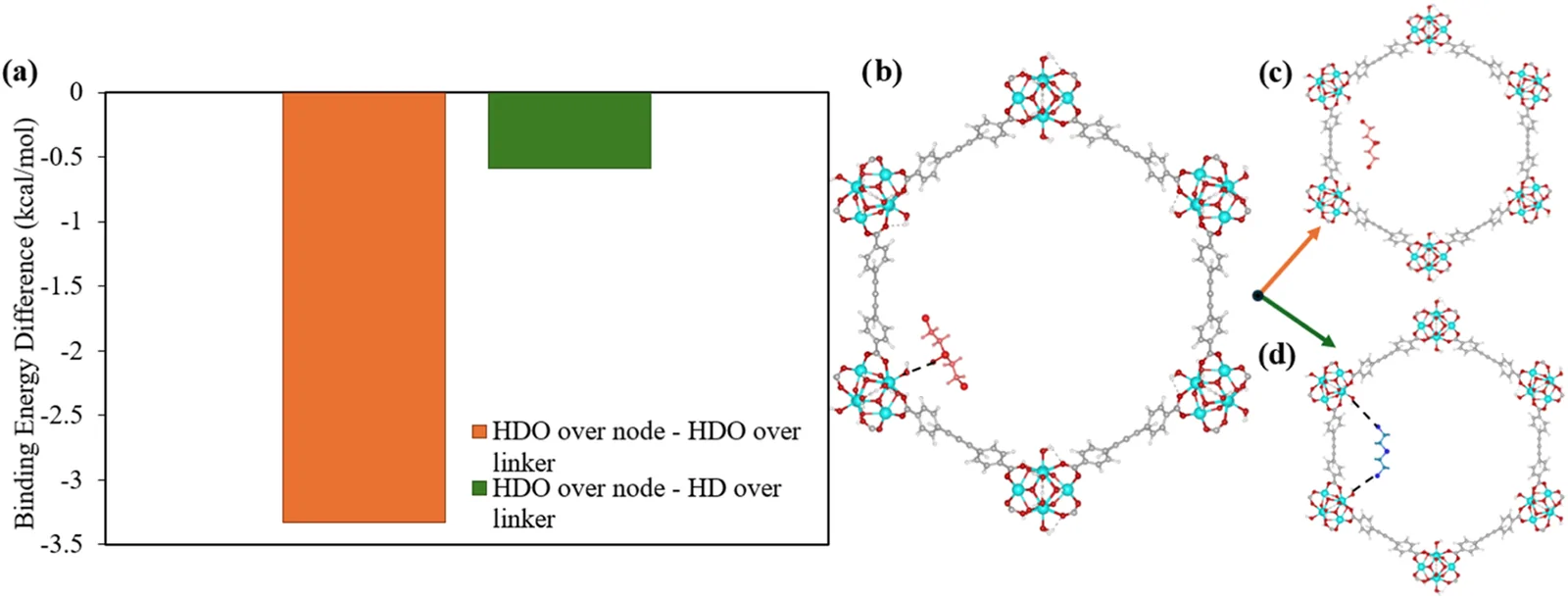
Mesopore binding interactions. (a) A comparison of HDO binding over the node vs HDO binding over organic linker. The more negative value of HDO over node represents a more favorable binding. (b) Geometry optimized HDO located over metal node in mesopore. (c) Geometry optimized HDO over the organic linkers in the mesopore. (d) Geometry optimized HD over the organic linkers, between two nodes.

The binding energy calculations provide a thermodynamic picture of local host–guest stabilization, whereas the MD simulations capture the combined effects of pore accessibility, guest motion, steric confinement, and guest–guest interactions at the selected low-loading conditions. Therefore, the agreement between DFT-preferred configurations, RDF interaction peaks, pore-occupancy analysis, and MSD-derived diffusion coefficients provides a consistent molecular picture of HD/HDO differentiation in NU-1000. Overall, HD exhibits higher binding energy values within NU-1000 in the smallest c-pore channels. This agrees with the MSD and RDF values where HDO exhibits a more diffuse profile and HD tends to maintain a more preferential pore location with increased interactions with the linkers from a constrained environment, hydrated Zr sites, and μ3-OH sites. While HDO does exhibit favorable interactions with H_2_O–Zr sites according to the RDF and mesopore preferential location binding energies, HDO has a very direct form of contact with the NU-1000 structure. In contrast HD exhibits strong van der Waals and hydrogen–halogen interactions because of its nonpolar, linear structure. These interactions were deduced from energy dependent calculations providing a clearer understanding of HDO preferential location near the metal sites and HD preferential interactions stretching across the organic linkers.^67^ This distinction again reinforces the role of NU-1000 in promoting spatial separation of reactant and product species, ultimately enabling selective transport.

## 4 Conclusion

Zr-MOFs have already been established as promising platforms for the capture and detoxification of chemical warfare agents, and NU-1000 is especially attractive because it combines structural robustness, photoactive pyrene linkers, hydrated Zr nodes, and a hierarchical pore network. The key question addressed in this work is therefore not whether NU-1000 is a promising member of the Zr-MOF family, but how its internal micropore, mesopore, and c-pore environments control the relative residence, interaction modes, and transport of sulfur mustard (HD) and its partially oxidized product mustard sulfoxide (HDO). By combining molecular dynamics simulations, Monte Carlo adsorption calculations, radial distribution function analysis, and DFT binding-energy calculations, this study identifies NU-1000 as a dual-function molecular host that differentiates reactant and product species through internal interface chemistry and confinement.

The Monte Carlo adsorption calculations show comparable saturated loadings of HD and HDO in NU-1000, indicating that total adsorption capacity alone does not explain their distinct behavior. Instead, the decisive difference arises from pore-selective residence and transport. At comparable low loading, HD localizes predominantly in microporous regions, occupying this region for approximately 80% of the trajectory, while HDO samples larger pore environments more broadly, with approximately 45% mesopore and 34% c-pore occupancy. This pore differentiation is preserved under mixed HD/HDO coadsorption conditions, showing that the observed reactant–product separation is intrinsic to the NU-1000 pore network rather than an artifact of isolated single-component simulations.

The molecular origin of this behavior is the different balance of dispersion stabilization, confinement, polarity, and node accessibility experienced by HD and HDO. DFT calculations show that HD binds most favorably in the c-pore region, with a binding energy of approximately −12.5 kcal/mol, and similarly favorable stabilization in micropore environments, whereas much weaker binding is observed in the mesopore. Together with RDF analysis, these results indicate that HD is stabilized by dispersion-dominated interactions with linker-rich confined environments and weak halogen–hydrogen contacts with hydrated node sites. In contrast, HDO exhibits more localized and orientation-dependent interactions involving the sulfoxide group and hydrated zirconium node environments. These interactions favor larger pore regions that provide sufficient configurational freedom, leading to broader spatial sampling and faster transport.

This dual-hosting behavior produces a pronounced transport asymmetry. HDO exhibits a self-diffusion coefficient of 10.6 ± 2.11 × 10^–10^ m^2^/s, compared with 0.5 ± 0.09 × 10^–10^ m^2^/s for HD at comparable loading. Thus, NU-1000 couples reactant retention with product mobility: the hydrophobic reactant is preferentially stabilized in confined micropore/c-pore environments, while the more polar oxidized product accesses mesopore and c-pore regions and diffuses more rapidly. This behavior provides a molecular-level basis for intrinsic reactant–product partitioning in a single hierarchical MOF framework (Table 1).

**Table 1:** Nonbonded Force Field Parameters for HD

Atom Type	ε (kcal/mol)	σ (Å)	*q*(*e*)
H1	0.0152	2.808	0.0375
H2	0.0152	2.808	0.0449
S	0.3440	3.531	–0.0180
Cl	0.2833	3.509	–0.0872
C1	0.0951	3.466	–0.0552
C2	0.0951	3.466	–0.0134

Overall, these findings show that the design value of NU-1000 extends beyond high uptake or catalytic activity alone. Its hierarchical pore structure creates chemically and spatially distinct internal interfaces that can differentially host a reactant and product according to their polarity, geometry, and preferred interaction modes. This work highlights the importance of considering pore-resolved residence, host–guest interaction specificity, and diffusion asymmetry when designing MOF-based systems for chemical warfare agent detoxification and related confined separation or catalytic processes. Future studies that combine finite subvolume diffusion analysis, loading dependent coadsorption, and experimental validation will further clarify how this dual-hosting mechanism translates across operating conditions and related Zr-MOF platforms (Table 2).

**Table 2:** Nonbonded Force Field Parameters for HDO

Atom Type	ε (kcal/mol)	σ (Å)	*q*(*e*)
O	0.0957	3.020	–0.3945
H1	0.0152	2.808	0.0525
H2	0.0152	2.808	0.0484
S	0.3440	3.531	0.2607
Cl	0.2833	3.509	–0.0523
C1	0.0951	3.466	–0.0601
C2	0.0951	3.466	–0.0225

## Supplementary Material



## Data Availability

The data supporting this article have been included as part of the Supporting Information (SI). Supporting Information: (a) a model LAMMPS input script and data file. (b) The self-diffusion coefficients from different loadings of HDO in NU-1000 at 300 K. (c) The trajectory video and the snapshots from 5 HDO and 25 HDO in NU-1000. (d) Directional diffusion coefficients for HD in NU-1000. (e) Charge model MD analysis with MSD. (f) Fickian diffusion regime justification. (g) The binding energies for Figures 8 and 9 of the main text. (h) S=O moiety charge model literature comparison. (i) The VASP geometry optimization INCAR.

## Notes

Directional components of HD MSD within NU-1000; analysis of DMol3 charge models Mulliken and Hirshfeld for both HD and HDO in independent trajectory analysis NU-1000 (ZIP)
