# Supplementary material for: Interfacial Confinement Enables Dual Hosting of Sulfur Mustard and Mustard Sulfoxide in NU-1000
Source: Langmuir. 2026 Oct 10;42(41):30833–46. doi: 10.1021/acs.langmuir.6c04057 (PMC13689922; doi:10.1021/acs.langmuir.6c04057)
Supplement: Supplementary file 1 [file la6c04057_si_001.zip › SI/SI.docx]

**Supplementary Information:**

**Interfacial Confinement Enables Dual Hosting of Sulfur Mustard and Mustard Sulfoxide in NU-1000**

Farrah S. McCormick^a^ and Liangliang Huang^a,b^*

a. School of Sustainable Chemical, Biological and Materials Engineering, University of Oklahoma, Norman, Oklahoma 73019, United States

b. Materials Science and Engineering Program, University of Oklahoma, Norman, Oklahoma 73019, United States

Self Diffusion Coefficient (10-10 m2/s)

| Number of HDO molecules in NU-1000 | Diffusion (x10 ^-10) |
| --- | --- |
| 5 | 1.84 |
| 10 | 2.64 |
| 15 | 3.87 |
| 20 | 8.9 |
| 25 | 10.4 |

**Figure S-1**: Self- Diffusion Coefficients calculated from different loadings of HDO in NU-1000 at 300K.

| **a)**  | **b)**  |
| --- | --- |

**Figure S-2**: Trajectory snapshots from the varied loading calculations. a) 5 HDO molecules in NU-1000. b) 25 HDO molecules in NU-1000.

HD Self Diffusion Rates (10-11 m2/s)

|  | Dx | Dy | Dz |
| --- | --- | --- | --- |
| 1 | 4.08 | 4.47 | 10.1 |

**Figure S-3**: Directional components of HD MSD within NU-1000.

Self Diffusion Coefficient of Different Charge Paramters for HD and HDO (m2/s)

|  | HD Mulliken | HD Hirshfeld | HDO Mulliken | HDO Hirshfeld |
| --- | --- | --- | --- | --- |
| 1 | 2.20087e-11 | 5.34586e-11 | 6.98e-10 | 1.06159e-09 |

**Figure S-4:** Analysis of DMol3 charge models Mulliken and Hirshfeld for both HD and HDO in independent trajectory analysis NU-1000.

**Figure S-5:** Alpha analysis for Fickian long-time diffusion regime. Section 2.2.1 in main text.

**Table 1**: DFT and MD binding energy values for Figure 8 and Figure 9 within the main text.

| **Pore Location** | **HD Binding Energy (kcal/mol) DFT** | **HD Binding Energy (kcal/mol) MD** | **HDO Binding Energy (kcal/mol) DFT** | **HDO Binding Energy (kcal/mol) MD** |
| --- | --- | --- | --- | --- |
| C-Pore | -12.5113 (Figure 8b) | -10.8847 | -10.5343 (Figure 8c) | -8.5542 |
| Micropore | -11.9676 (Figure 8d) | -13.6381 | -10.0160 (Figure 8e) | -9.9272 |
| Mesopore | -0.9097 (Figure 9d) | -1.0420 | -1.4959 (Figure 9b) | -3.4398 |

**Table 2**: S=O moiety charge analysis comparison with literature DMSO values.

| Charge Model | S Charge | O Charge | ∆q(S-O) |
| --- | --- | --- | --- |
| *HDO Hirshfeld* | +0.261 | -0.395 | 0.656 |
| *HDO Mulliken* | +0.194 | -0.518 | 0.712 |
| *DMSO Bordat FF*^1^ | +0.117 | -0.437 | 0.554 |
| *DMSO Liu FF*^2^ | +0.139 | -0.459 | 0.598 |
| *DMSO CHELPG*^3^ | +0.343 | ~-0.56 | ~0.90 |
| *DMSO MK ESP*^3^ | +0.410 | ~-0.56 | ~0.97 |
| *DMSO Mulliken*^3^ | +0.725 | ~-0.56 | ~1.29 |

Example Geometry Optimization Input VASP Script

SYSTEM = NU-1000

PREC	= Normal

ENCUT	= 520

EDIFF	= 1E-6

EDIFFG	= -0.05

ISMEAR = 0

SIGMA	= 0.05

IBRION	= 2

NSW	= 400

ISIF	= 2

LASPH	=. TRUE.

ADDGRID =. TRUE.

LREAL = Auto

ALGO = Normal

IVDW	= 12

NELM	= 300

References

(1)	Bordat, P.; Sacristan, J.; Reith, D.; Girard, S.; Glättli, A.; Müller-Plathe, F. An Improved Dimethyl Sulfoxide Force Field for Molecular Dynamics Simulations. *Chem. Phys. Lett.* **2003**, *374* (3–4), 201–205. https://doi.org/10.1016/S0009-2614(03)00550-5.

(2)	Liu, H.; Mueller-Plathe, F.; Van Gunsteren, W. F. A Force Field for Liquid Dimethyl Sulfoxide and Physical Properties of Liquid Dimethyl Sulfoxide Calculated Using Molecular Dynamics Simulation. *J. Am. Chem. Soc.* **1995**, *117* (15), 4363–4366. https://doi.org/10.1021/ja00120a018.

(3)	Wiewiór, P. P.; Shirota, H.; Castner, E. W. Aqueous Dimethyl Sulfoxide Solutions: Inter- and Intra-Molecular Dynamics. *J. Chem. Phys.* **2002**, *116* (11), 4643–4654. https://doi.org/10.1063/1.1449864.
